# Supplementary material for: Mutation-specific reporter for optimization and enrichment of prime editing
Source: Nat Commun. 2022 Mar 1;13:1028. doi: 10.1038/s41467-022-28656-3 (PMC8888566; doi:10.1038/s41467-022-28656-3)
Supplement: Supplementary file 1 — Supplementary Information [file 41467_2022_28656_MOESM1_ESM.pdf]

Supplementary Information

Supplementary Figures 1-12
Supplementary Table 1: off-target analyses in fluoPEER-edited cells
Supplementary Note 1: FluoPEER design and plasmid cloning
Supplementary References

a

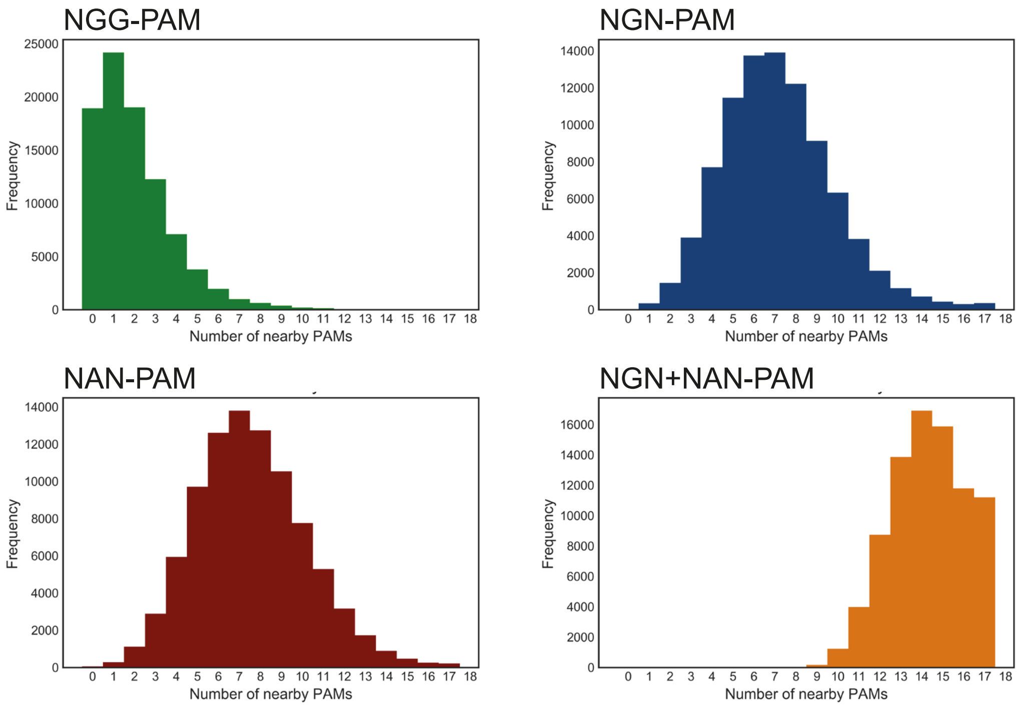

b

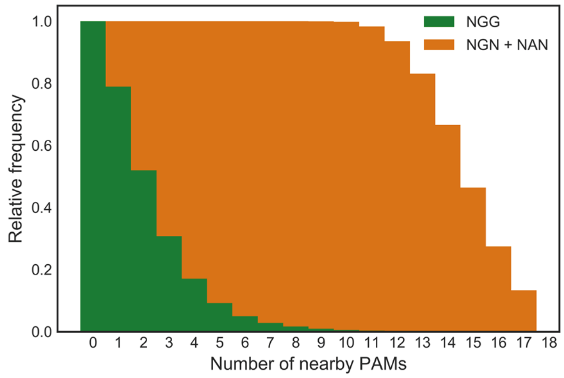

Supplementary Figure 1: Analysis of the targeting scope of (NGG-)PE2 and flexible PE2 variants within the ClinVar database

a, The number of NGG-, NAN-, or NGN-PAMs within the genomic region surrounding pathogenic variants in the ClinVar database. Only mutations shorter than 51 bp were considered as correctable by prime editing and PAM sites in a window of 10 bp upstream to 4 bp downstream of the target site were considered usable, based on Kim et al. (2021)[1].

b, Cumulative histogram of the number of available PAM sites shows that flexible PE2 variants (NGN+NAN) have at least 10 PAM sites available for the repair of pathogenic variants in the ClinVar database.

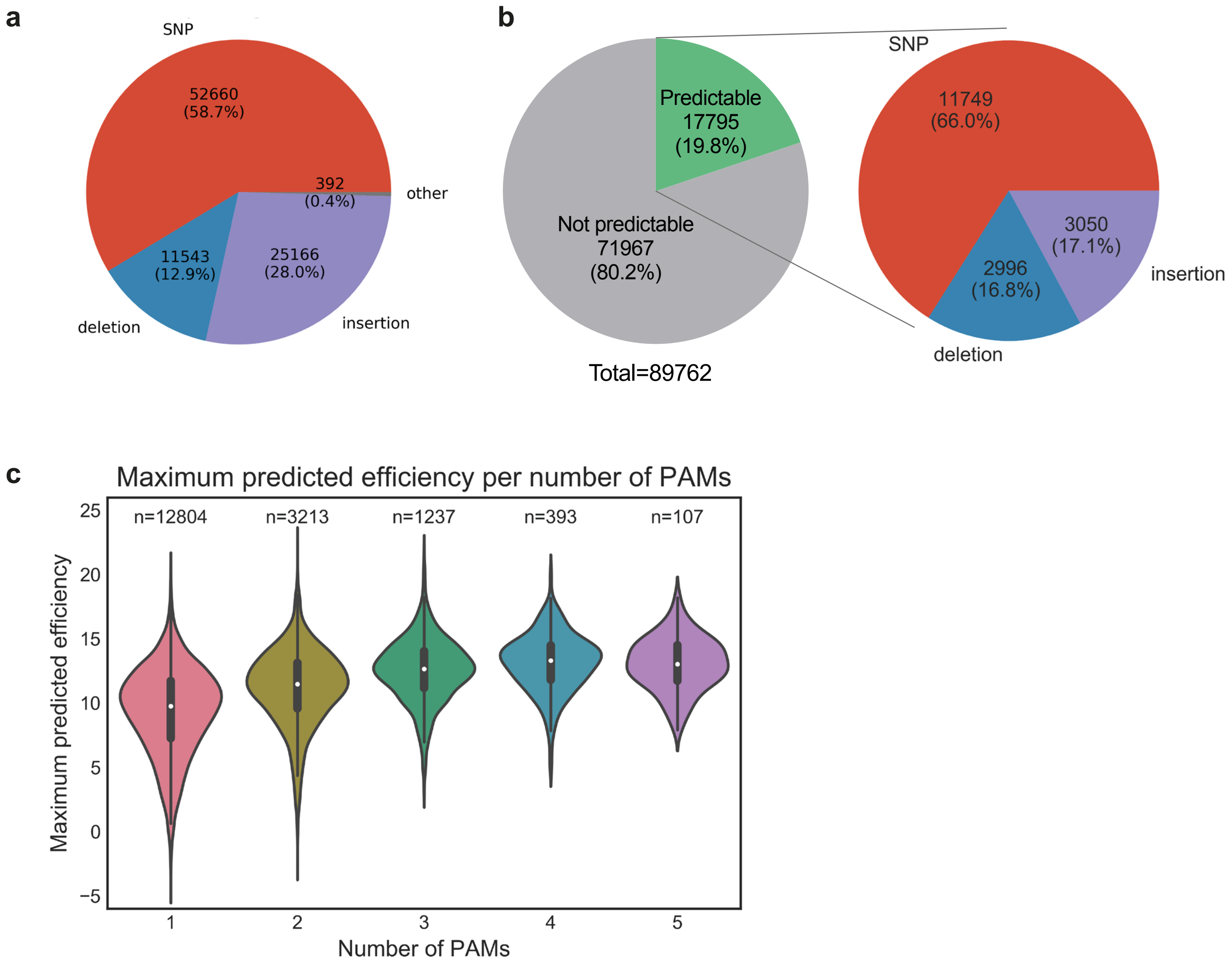

Supplementary Figure 2: Evaluation of reparable mutations in the ClinVar database within the scope of the PE_prediction machine learning algorithm

a, Types of mutations of pathogenic variants in the ClinVar database.

b, Number (left panel) and type (right panel) of pathogenic variants in the ClinVar database for which the PE_prediction machine learning algorithm can predict the efficiency of a mutation-correcting pegRNA.

c, Maximum predicted efficiency for repair of ClinVar pathogenic variants for mutations that fall within the scope of the DeepPE deep learning algorithm. Increase in available PAMs for pathogenic variants results in a higher median maximum predicted efficiency. Violin plots indicate the median (white dot), the interquantile range (thick gray bar), and the complete distribution excluding outliers (thin gray line).

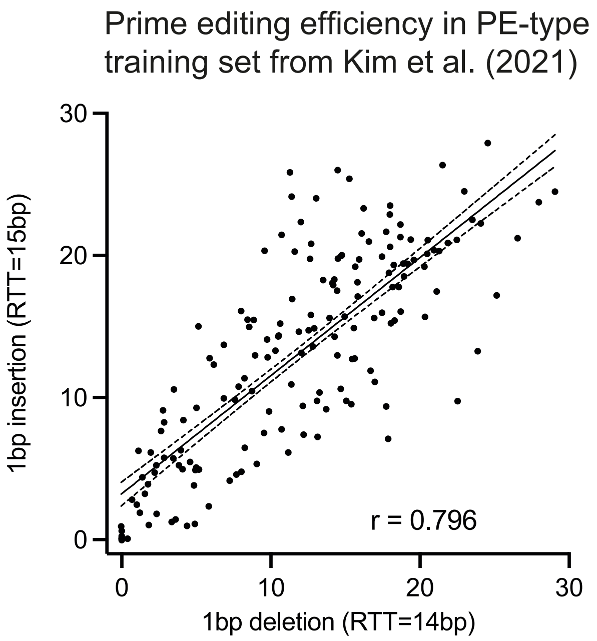

Supplementary Figure 3: Correlation of prime-editing efficiency of 1-bp deletions versus 1-bp insertions

Re-analysis of data from Kim et al. (2021)[1] to estimate the correlation of prime editing efficiencies of pegRNAs that create a 1-bp deletion (x-axis) to the efficiencies of corresponding pegRNAs with the same spacer and PBS sequence that create a 1-bp insertion. 1-bp substitution-edits could not be included in this comparison since Kim et al. performed 1-bp substitutions with much longer RTTs than 1-bp deletions. Since generation of these 1-bp deletions and insertions only involve a subtle difference in the RTT, optimal prediction efficiency would be reflected by r=1. Source data are provided as a Source Data file.

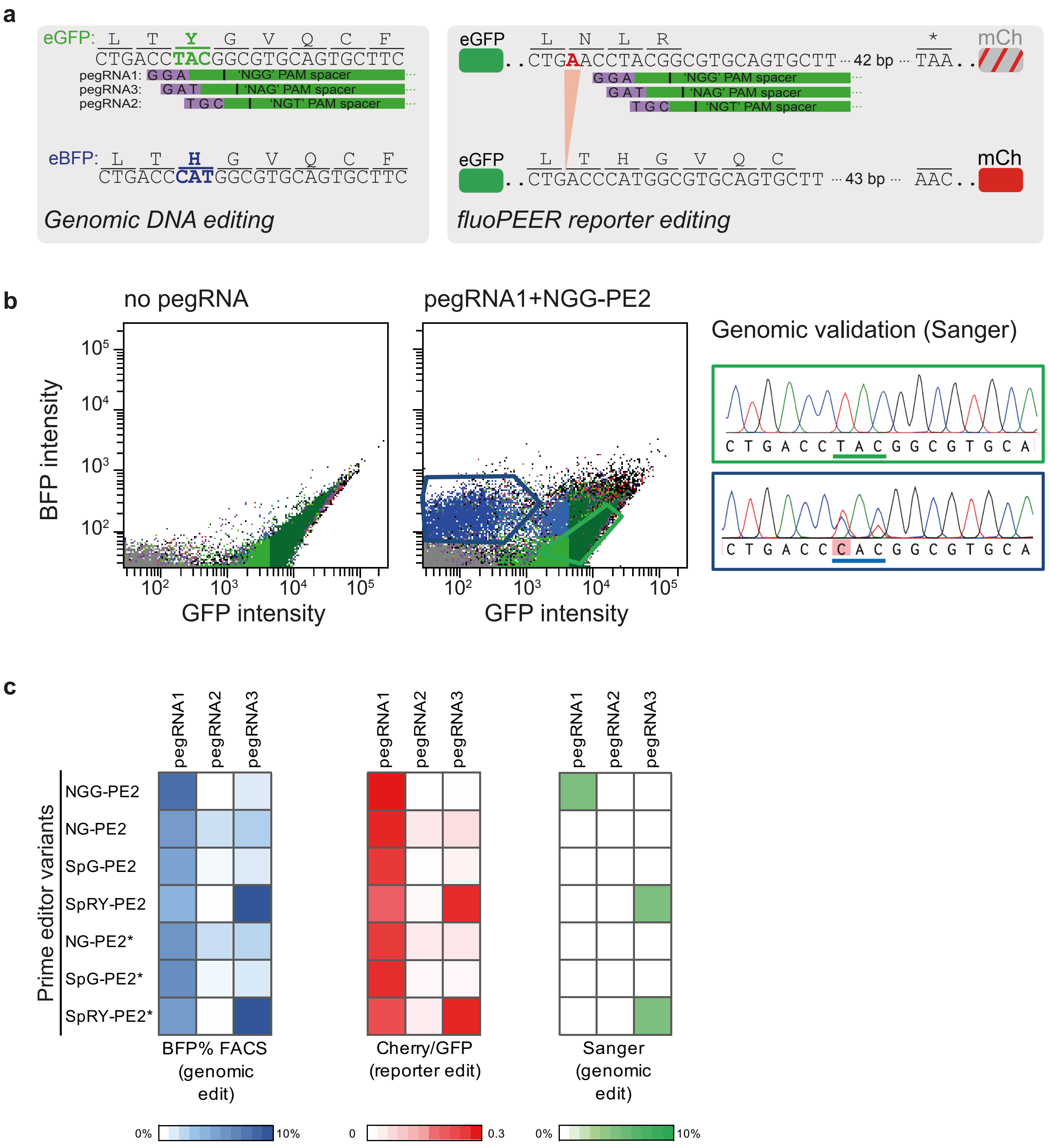

Supplementary Figure 4: Comparison of genomic editing of *GFP→BFP* to editing on the corresponding fluoPEER plasmid

a, Design of three different pegRNAs converting *GFP* to *BFP* (TAC to CAT). The same three pegRNAs remove a single nucleotide insert on the corresponding fluoPEER plasmid, resolving a frameshift mutation upstream of the Cherry cassette. Note that the PAM sequences (purple) of the pegRNA 1, 2, and 3 are NGG, NGN, and NAN, respectively.

b, HEK293T cells containing a lentivirally integrated genomic GFP cassette were transfected with the prime editing machinery to convert GFP to BFP. FACS plot shows occurrence of BFP+ cells 14 days after transfection with pegRNA1 (NGG-PAM) and NGG-PE2. Sanger sequencing confirms TAC to CAT conversion in BFP+ cells (blue outlines), but not in GFP+ cells (green outlines).

c, Editing of integrated genomic GFP (left panel) and editing of the corresponding fluoPEER plasmid (middle panel) in HEK293T cells using the three pegRNA designs from (a) and 7 different prime editor variants (all flexible PAM prime editor variants and the NLS-adjusted 'PE2*' versions from Liu et al.[3]). Sanger sequencing of the genomic target region (right panel) in the same cells as shown in the left panel illustrates that Sanger is not sensitive enough to quantify *GFP* to *BFP* conversion for most prime editing conditions. When less than 10% of cells (FACS data) are edited, Sanger sequencing could not quantify any specific editing. Related to figure 1d. Source data are provided as a Source Data file.

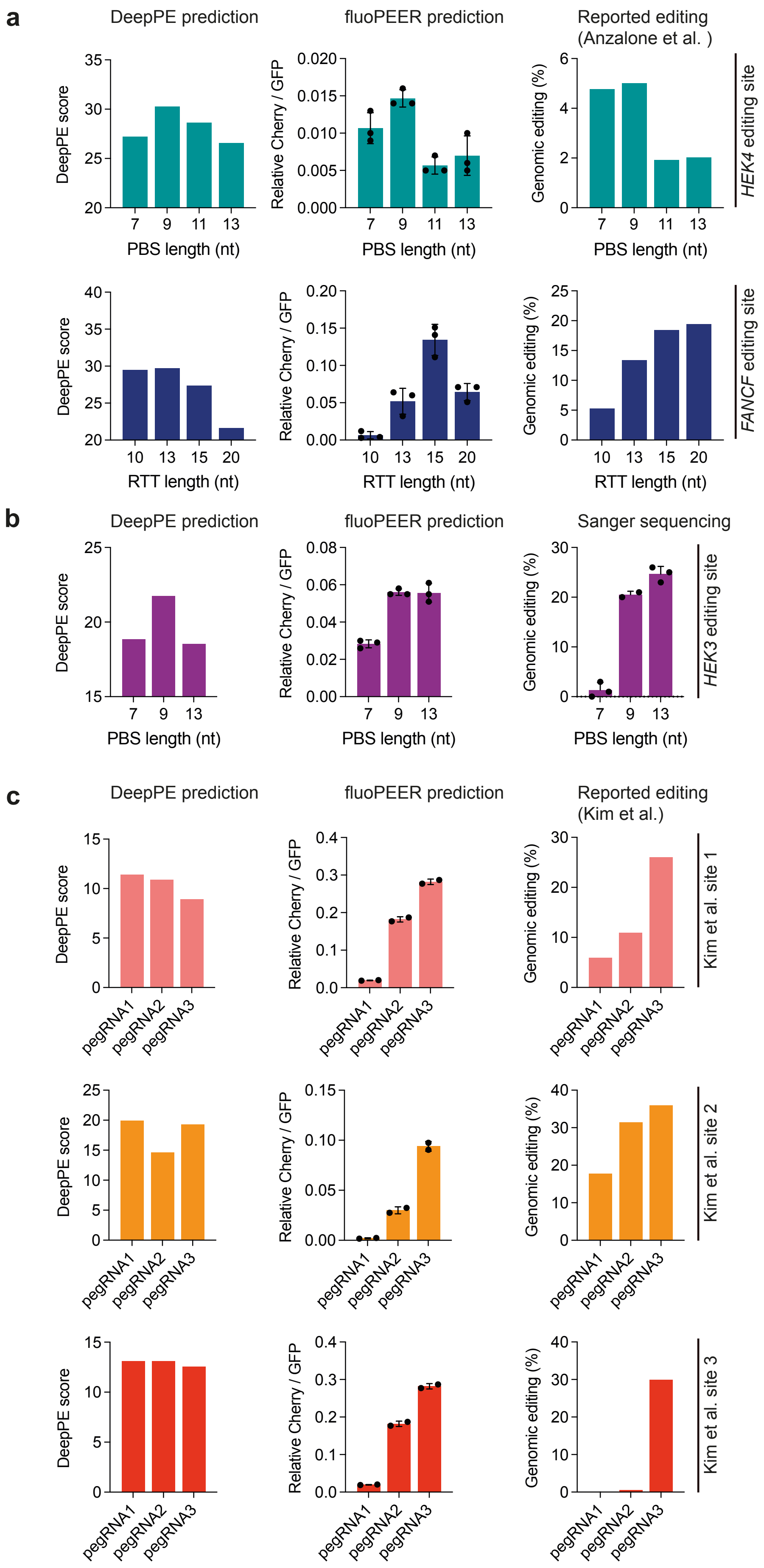

Supplementary Figure 5: Comparison of fluoPEER ranking with predictions by the DeepPE algorithm

Comparison of the prediction score of the DeepPE algorithm (left panels), fluoPEER-derived efficiency ratio (Cherry/GFP; middle panel), and the actual prime editing efficiencies as reported by **(a)** Anzalone et al. (2019)[2] **(b)** Sanger sequencing performed for this work, and **(c)** Kim et al. (2021)[1]. Source data are provided as a Source Data file.

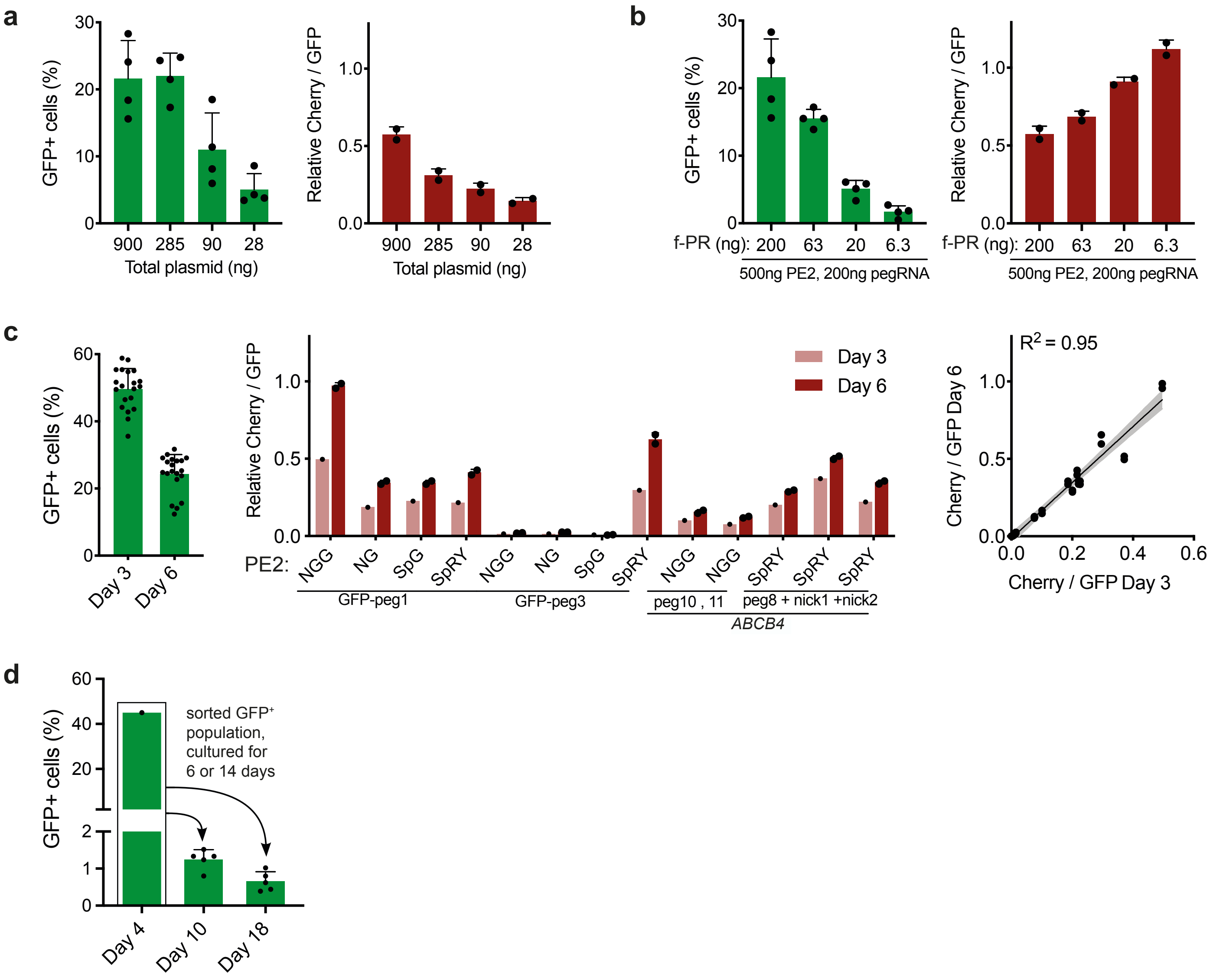

Supplementary Figure 6: Dynamics of fluoPEER under various transfection conditions and read-out times

a, Percentage of GFP+ cells and average of Cherry to GFP ratio in GFP+ HEK293T cells with decreasing amounts of total transfected plasmid DNA (PE2+pegRNA+fluoPEER).

b, Percentage of GFP+ cells and average of Cherry to GFP ratio in GFP+ HEK293T cells with decreasing amounts of the fluoPEER (f-PR) plasmid DNA but equal amounts of PE2 and pegRNA plasmid DNA. As expected, lowering fluoPEER concentration led to decreased percentages of GFP+ cells, but increased reporter editing (Cherry to GFP ratio).

c, Percentage of GFP+ cells and average of Cherry to GFP ratio in transfected cells, as measured 3 and 6 days after transfection of PE2+pegRNA+fluoPEER.

d, Percentage of GFP+ cells after transfection (Day 0) of the fluoPEER plasmid. On day 4, GFP+ (transfected) cells were FACS sorted to get a 100% GFP+ HEK293T population. These cells were plated and subsequently FACS sorted on day 10 and day 18 to measure disappearance of GFP-positivity. Source data are provided as a Source Data file. Error bars represent standard deviations from the mean of at least *n* = 2 biologically independent replicates.

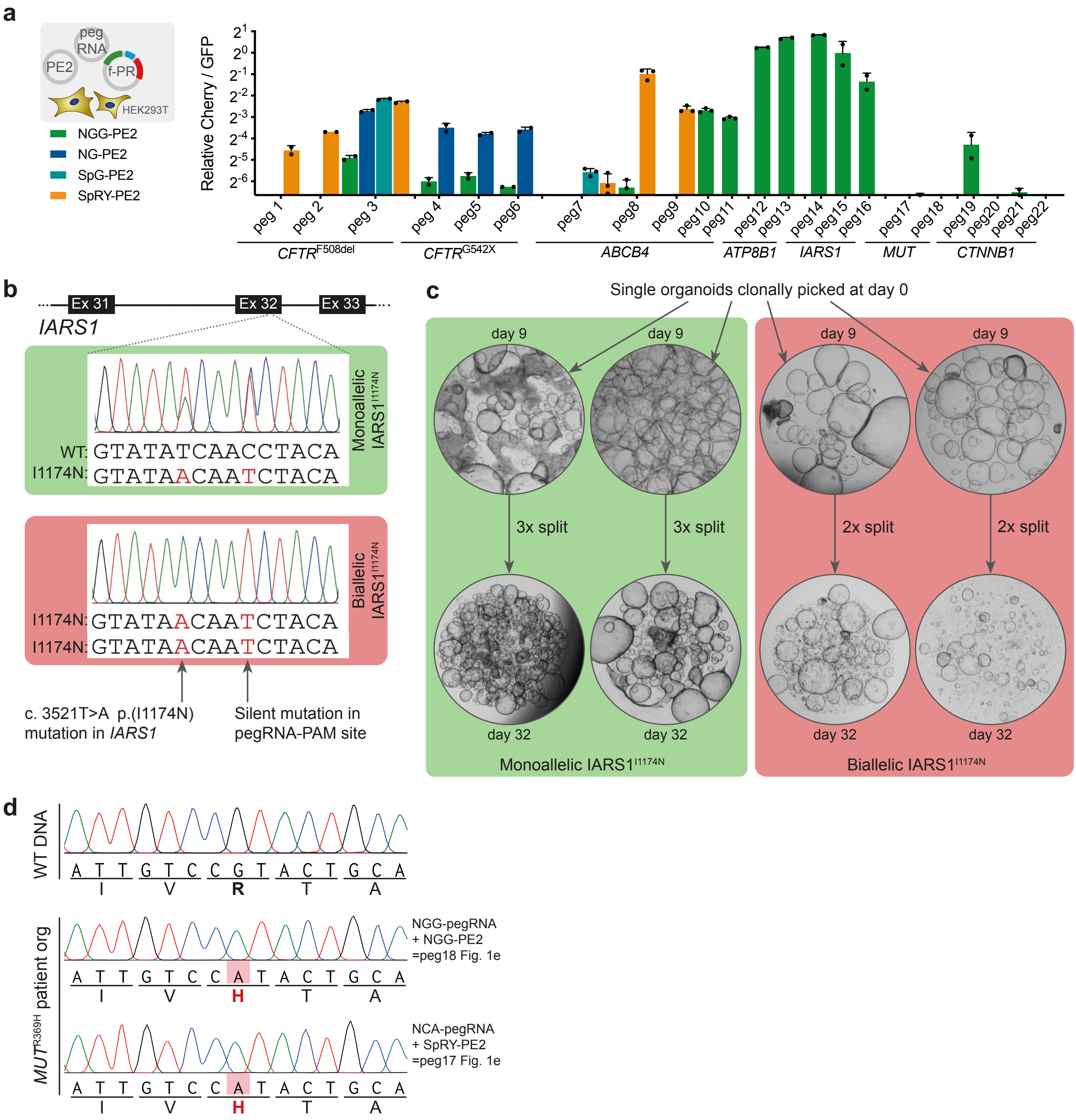

Supplementary Figure 7: testing various pegRNAs and prime editor variants for seven genomic loci and fluoPEER-predicted editing in organoids

a, FluoPEER plasmids were created to test prime editing strategies for the correction or creation of seven different genomic mutations. FluoPEER plasmids were transfected into HEK293T cells together with the indicated pegRNA + prime editor combinations. Cherry over GFP signal ratio was measured using FACS analysis after 3 days. Related to Fig. 1f, in which the pegRNA + prime editor combinations with the highest fluoPEER prediction scores were used for prime editing in primary organoid cells. Error bars represent standard deviations from the mean of at least *n* = 2-3 biologically independent replicates.

b, Sanger sequencing of clonal liver-derived organoids with monoallelic and biallelic I1174N mutations in exon 32 of *IARS1*, using peg14 from (a).

c, Clonal organoid lines with monoallelic (2 clones) and biallelic (2 clones) *IARS1*^{I1174N} mutations were continually passaged for 32 days. Clones with biallelic, but not monoallelic, *IARS1*^{I1174N} mutations had lower organoid reconstitution capacity and could be passaged less often due to slower growth.

d, Correction of pathogenic *MUT*^{R369H} mutations in liver organoids derived from an MMA patient could not be corrected using two different pegRNA designs. This corresponds to very low fluoPEER prediction scores for these targeting strategies as shown in (a). Source data are provided as a Source Data file.

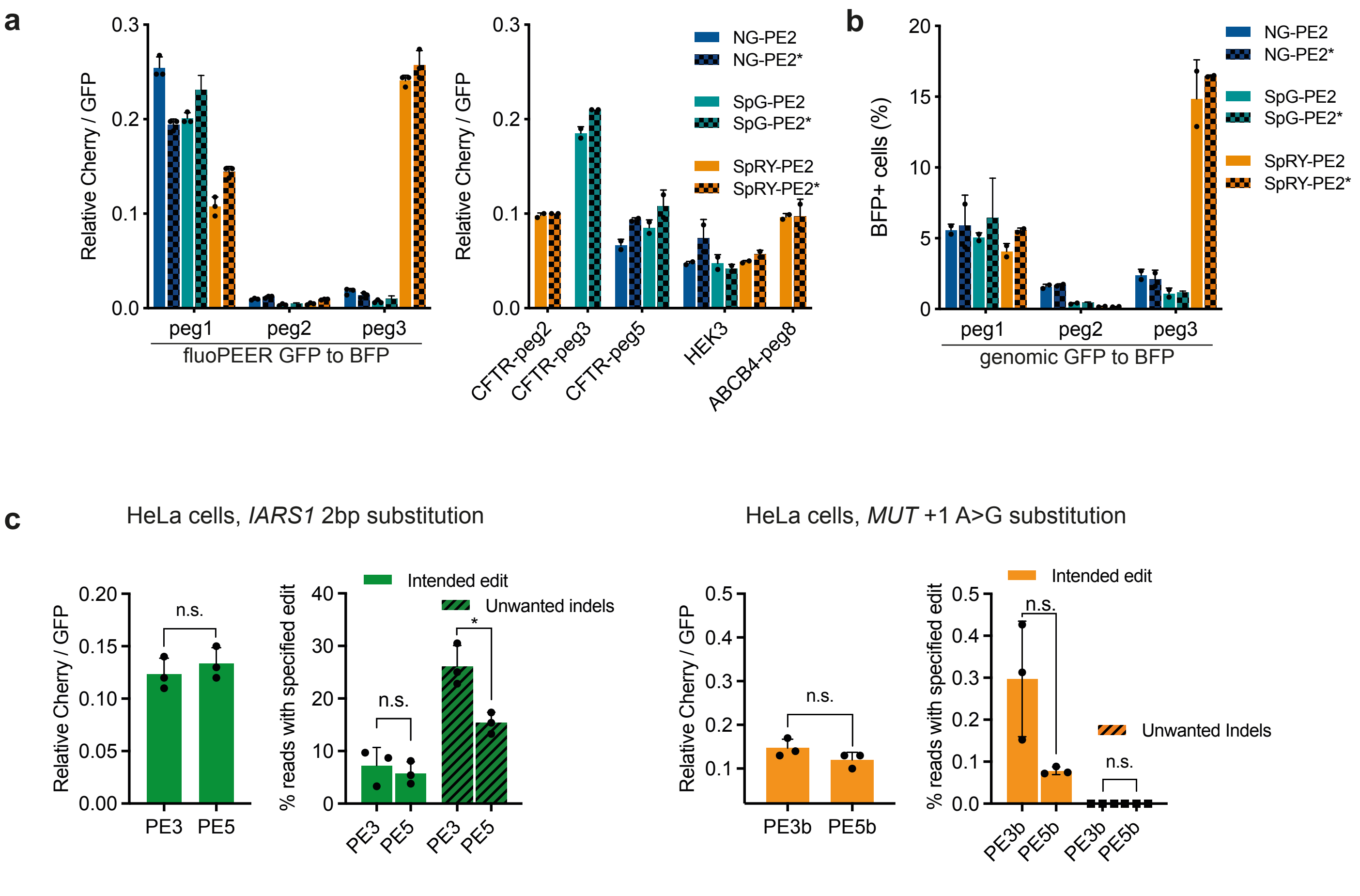

Supplementary Figure 8: Characterization of prime editor NLS adaptations and PE4max using fluoPEER

a, Comparison of fluoPEER scores for flexible PE2 variants and the corresponding PE2* variants with nuclear localization sequence (NLS) adaptations[3].

b, Conversion of genomically integrated *GFP* to *BFP* using various PE2 and PE2* variants. Related to Fig. 2b. Note that the PE2* NLS adaptations did not result in an overall increase of fluoPEER scores nor an overall increase in genomic editing efficiency. The data shown in Supplementary Fig. 4c were reused for (b).

c, Comparison of fluoPEER scores and genomic editing for PE3(b) (PE2 + nicking gRNA) and PE5(b) (PE4max + nicking gRNA). PE5(b) does not yield higher fluoPEER scores, nor higher genomic editing efficiencies for an *IARS1* 2-bp substitution and *MUT* 1-bp substitution (+1 A>G) mutation in HeLa cells compared to PE3(b). However, PE5 yields fewer unwanted indels compared to PE3 for the *IARS1* 2-bp substitution. Error bars represent standard deviations from the mean of at least n = 2-3 biologically independent replicates. Significance was analyzed using a two-tailed unpaired Student's t-test (*P <0.05). Source data are provided as a Source Data file.

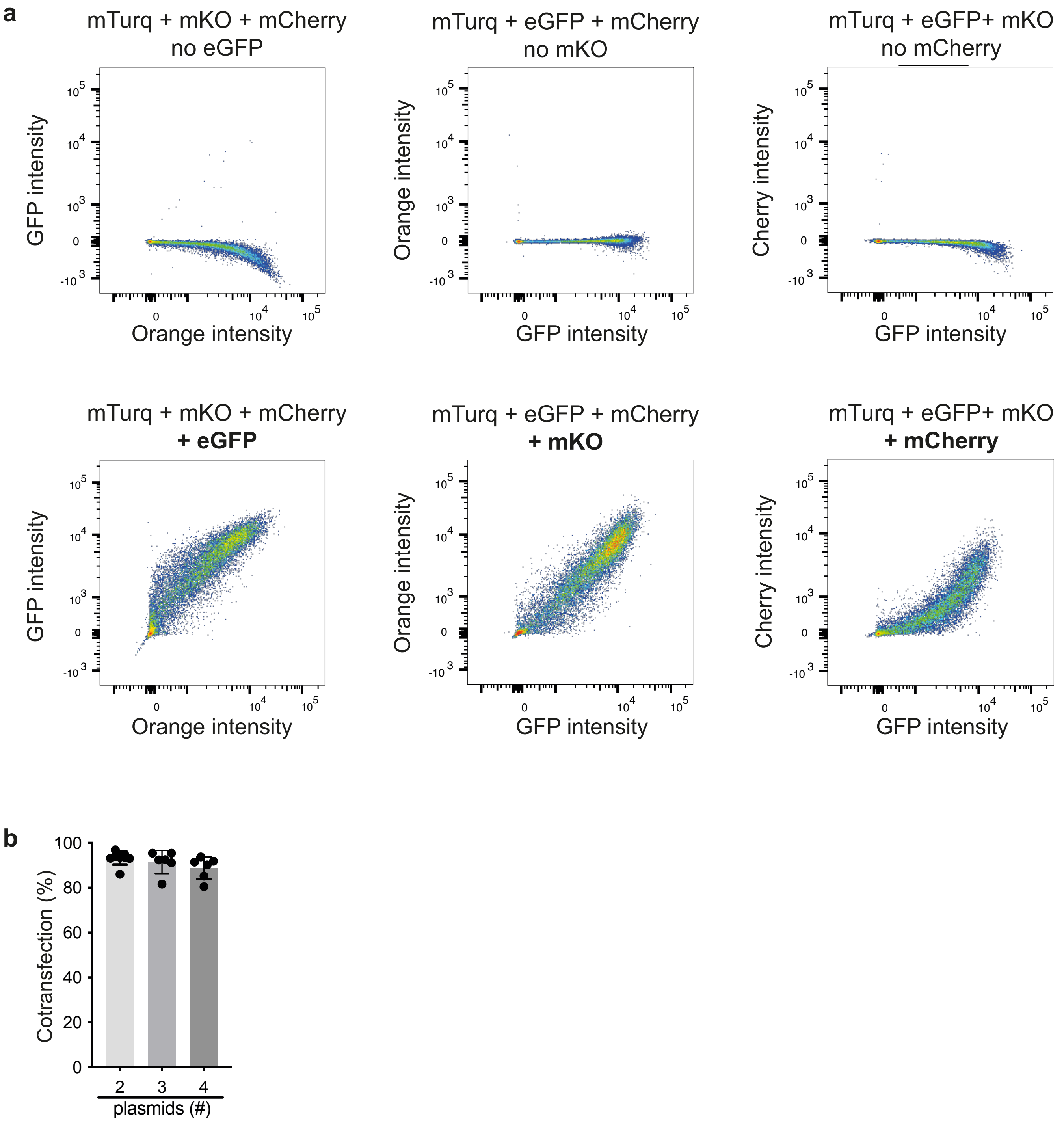

Supplementary Figure 9: Dynamics of cotransfection of four fluorescent plasmids

a, Mixtures of plasmids (5kb) containing CMV-mTurquoise2, CMV-eGFP, CMV-mKO2, and/or CMV-mCherry were transfected into HEK293T cells and inspected by FACS after 2 days. FACS plots show proper compensation in the 'fluorescence minus one' (FOI) controls (top row) and a strong correlation between fluorescent intensities from different plasmids in the condition containing all four plasmids (bottom row)

b, Quantification indicated very high (>90%) cotransfection efficiency. This strongly suggests that 2-4 fold enrichment of genomic editing in fluoPEER-edited vs. fluoPEER-unedited cells (Fig. 2a) cannot be the result of inefficient cotransfection of plasmids encoding prime editing machinery (PE2+pegRNA) in fluoPEER-unedited cells. Error bars represent standard deviations from the mean of *n* = 6 biologically independent replicates. Source data are provided as a Source Data file.

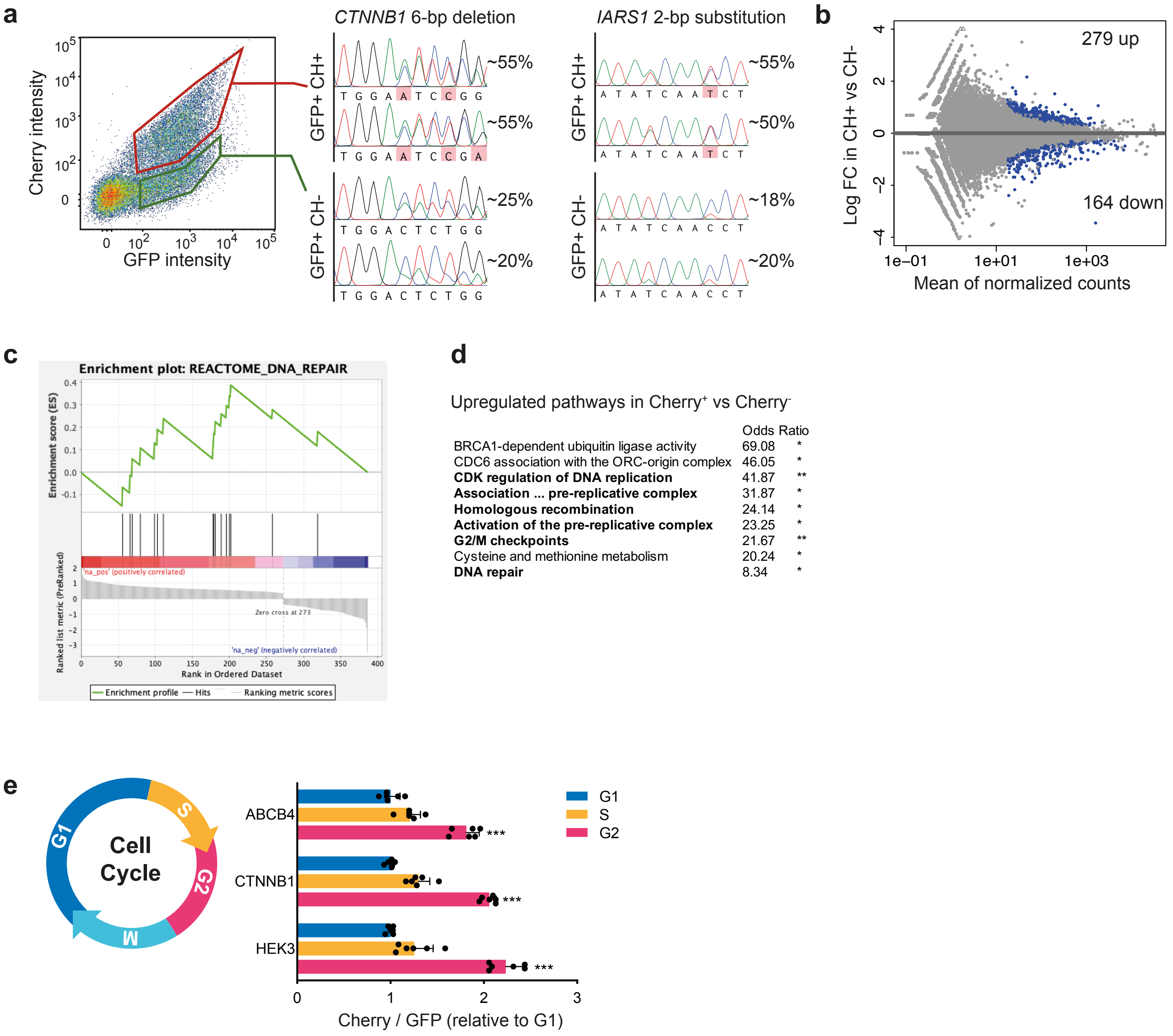

Supplementary Figure 10: RNA sequencing analysis of fluoPEER-edited versus -unedited cells

a, HEK293T cells were transfected with prime editor and pegRNA plasmids creating either *IARS1* 2-bp substitution or *CTNNB1* 6-bp deletion mutations, together with the corresponding fluoPEER plasmid. 48 hours after transfection, fluoPEER-edited (GFP$^+$CH$^+$) and fluoPEER-unedited (GFP$^+$CH$^-$) cells were sorted and genomic editing efficiencies for each population were estimated using Sanger sequencing.

b, RNA-sequencing was performed in replicates of each population from (a). MA-plot shows significant (adjusted *p*-value <0.05) up- and downregulation of 279 and 164 genes, respectively in fluoPEER-edited versus -unedited cells.

c, Enrichment plot of the 'DNA repair' pathway, which was the most enriched 'Reactome Database' pathway in fluoPEER-edited versus -unedited cells.

d, EnrichR analysis of fluoPEER-edited versus -unedited cells.

e, Hoechst staining in HEK293T cells transfected with the fluoPEER plasmid and prime editing machinery shows enrichment of reporter editing signal in G2, 24 hours after transfection. Gating strategies can be found in Supplementary fig. 12b. Error bars represent standard deviations from the mean of n = 6 biologically independent replicates. Significance was analyzed using One-way ANOVA. Source data are provided as a Source Data file.

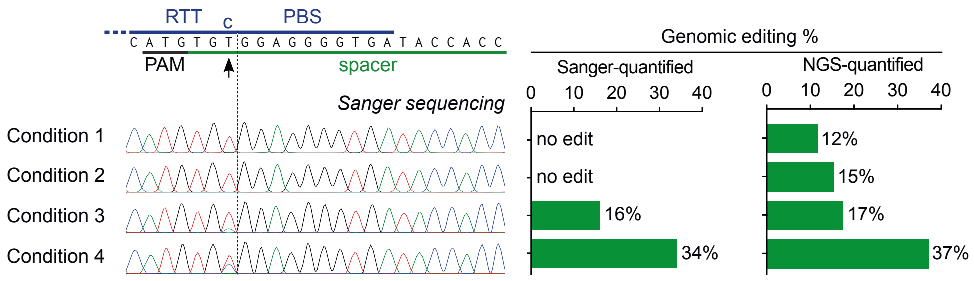

Supplementary Figure 11: Sanger sequencing is unable to quantify <15% editing of total reads
When comparing quantification of four prime editing conditions in HEK293T cells by Sanger sequencing and NGS, Sanger sequencing was unable to quantify edited reads when lower or equal to 15% of all reads. Source data are provided as a Source Data file.

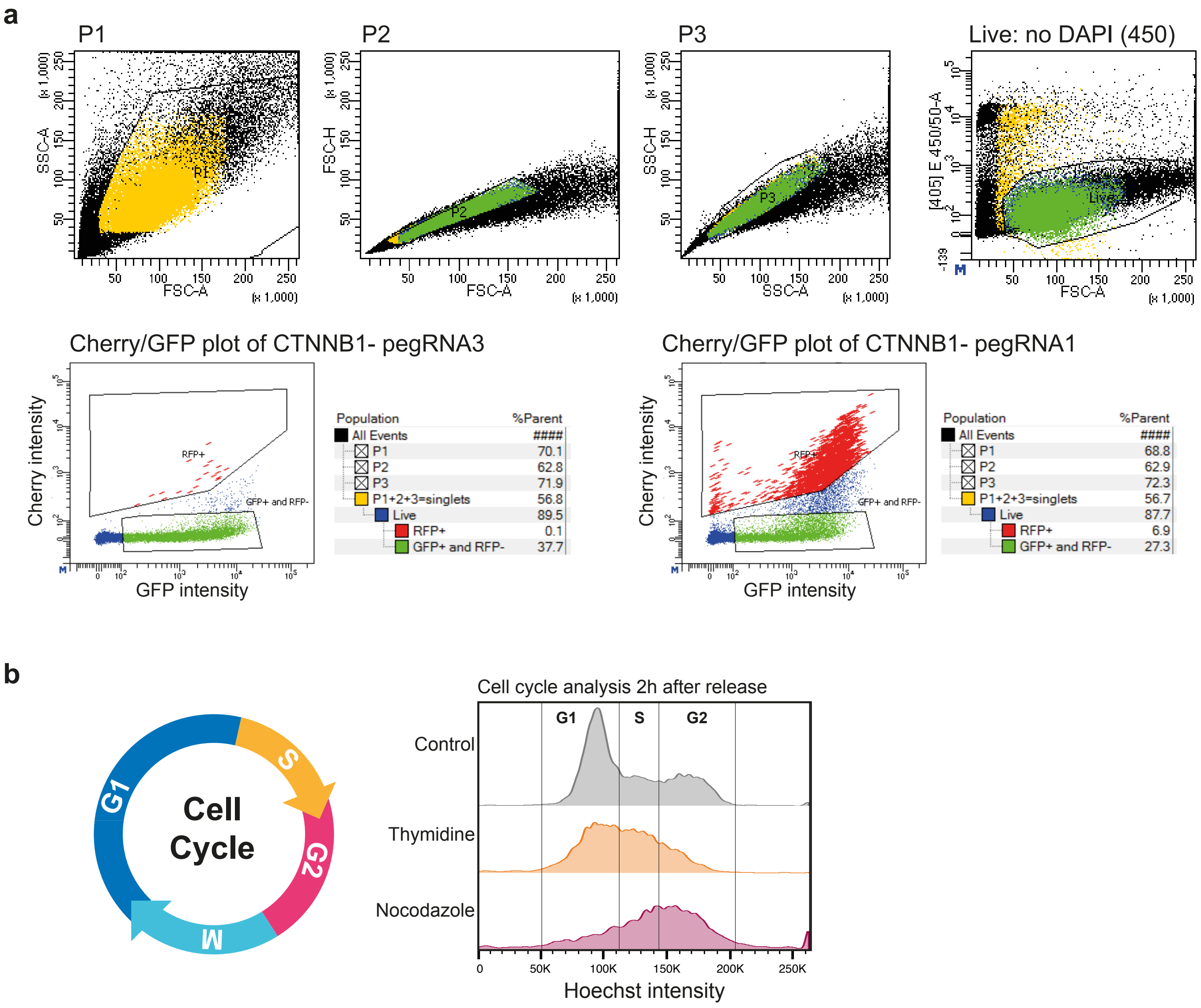

Supplementary Figure 12: Flow cytometry gating strategies

a, Example of gating strategy for a fluoPEER experiment in HEK293T cells. The 'P1+P2+P3' gate selects for single cells; the 'live' gate selects for DAPI negative, live cells. The GFP+ gate selects for cells that were transfected with fluorescent reporter plasmid. Lower left panel shows the Cherry/GFP plot and population hierarchy for an ineffective (*CTNNB1*, pegRNA1) and lower right panel for an effective (*CTNNB1*, pegRNA3) prime editing strategy.

b, Example of gating strategy for cell cycle phase based on Hoechst signal intensity. Note that cells were analyzed at the time of transfection of prime editing plasmids, which was 2 hours after release from G1/S or G2/M block.

Supplementary table 1: off-target analyses for two prime edit conditions in transfected cells vs. fluoPEER-edited cells.

Condition	pegRNA Spacer	Prime editor	Nearest off-target	Mismatches	Editing in negative control		Editing in transfected (GFP+)			Editing in fluoPEER-edited (GFP+RFP+)		
CTNNB1_5bp-del	CAACAGTCTTACCTGGACTC**NGG**	NGG-PE2	CA**G**CA**C**TCT**G**ACCTGGACTC**TGG**	3bp	0.0%	0.0%	0.0%	0.0%	0.0%	0.0%	0.0%	0.0%
MUT_+1 A>G	GGTGGTATCACCCCTCCACA**NNN**	SpRY-PE2	GGTGGTATCA**T**CCCTCC**T**CA**ATG**	2bp	0.1%	0.1%	0.1%	0.0%	0.0%	0.1%	0.0%	0.0%

No unwanted off-target editing was detected above the noise level (negative control) in any of the prime edit conditions.

Supplementary Note 1: FluoPEER design and plasmid cloning

pegRNA

PegRNAs should be designed according to the following guiding principles:

1. pegRNA spacers with high Cas9 sgRNA binding scores. Currently available Cas9 prediction algorithms have been developed using SpCas9 (NGG-recognizing), but do not discriminate between NGG and non-NGG PAMs. For this study, we selected spacers with an on-target score of at least 20, based on an algorithm developed by Doench et al. (2016)[3] which was employed in the Benchling web interface.
2. pegRNA primer binding sites (PBSs) with length 9-15 and a GC-count of at least 5 and a GC-content of at least 40%. If this is not possible, for instance when targeting a mutation in an AT-rich region, pick a PBS with a GC-count of at least 4 and GC-content >30%.
3. pegRNA reverse transcriptase templates (RTTs) with length 9-20, spanning the genomic region at least 6 bp downstream of the intended edit. For example, when the intended edit is located at position +6 from the pegRNA nicking site, the RTT should be at least 12 nucleotides in length. When the RTT length is less than 12, if possible, pick a G for the last edit.
4. If possible, the RTT should create a (silent) PAM mutation to increase editing efficiency. When using the flexible SpRY-PE2, PAM mutations are not expected to increase editing efficiency.

FluoPEER

fluoPEER needs a STOP codon or frameshift in the genomic insert to function. In case the genomic target already contains either, 45-100 bp (50-60 bp have been tested most) around the mutation of interest can be selected. The genomic insert should at least contain the full spacer and extension (PBS+RTT) sequences of all designed pegRNAs, including a margin of 5 bp upstream and 5 bp downstream. In case none such frameshift nor STOP codon is present, one will have to be inserted. In addition to the example provided in Fig. 1d and S4a, we will provide two examples below:

Example 1: *CFTR* 3bp insertion

The edit on the genomic DNA is a 3bp CTT insertion. This is not a frameshift mutation:

ATTAAAGAAAATATCAT---TGGTGTTTCCTATGATGAATATAGATAC →
ATTAAAGAAAATATCAT**CTT**TGGTGTTTCCTATGATGAATATAGATAC

For the fluoPEER, an additional T was inserted, leading to a frameshift mutation.

ATTAAAGAAAATAC**T**CAT---TGGTGTTTCCTATGATGAATATAGATAC →
ATTAAAGAAAATATCAT**CTT**TGGTGTTTCCTATGATGAATATAGATAC

Note that using this specific fluoPEER, only pegRNAs with spacers binding downstream from the 3bp insertion can be tested.

Example 2: *MUT* A>G point mutation

The edit on the genomic DNA is an A>G mutation, which does not resolve a STOP codon.

TCCCTTGGACGGCCAGATATTCTTGTCAAGTGTGGAGGGGTGATACCACCTCAG →
TCCCTTGGACGGCCAGATATTCTTGTCA**T**GTG**C**GGAGGGGTGATACCACCTCAG

For the fluoPEER, an additional A>t point mutation was added, leading to **STOP codon**.

TCCCTTGGACGGCCAGATATTCTTGTC**t**AGTGTGGAGGGGTGATACCACCTCAG →
TCCCTTGGACGGCCAGATATTCTTGTC**AT**GTG**C**GGAGGGGTGATACCACCTCAG

Important notes:

1. The fluoPEER plasmid and not the pegRNA design should be modified, such that the same pegRNAs can be used to edit both the fluoPEER plasmid and the genomic target.
2. In some cases, generating the desired mutation will create another stop downstream of the mutation (for example, when mutating at an exon-intron junction, this could occur). Always check the translation of the fluoPEER genomic insert before and after the intended edit for STOP codons; successfully edited inserts should not contain frameshifts or STOP codons.
3. The PAM site and spacer sequence (including PBS) of all pegRNAs should not be altered by the addition of a frameshift mutation or creation of a stop-mutation for the correct functioning of fluoPEER. In most cases, there is a significant difference in spacer binding scores and GC-content between the DNA sequence upstream and downstream of the target mutation, offering an obvious optimal region for spacer and PBS binding, respectively. This means that most of the tested pegRNAs will bind the same strand and can therefore be tested on the same fluoPEER plasmid. In case it is unavoidable, design two fluoPEER plasmids for the mutation instead.

STEP 1

Digest the pmGFP-P2A-K0-P2A-RFP plasmid by making the following mix:

Component	Volume
1 µg pmGFP-P2A-K0-P2A-RFP plasmid	X
SalI (NEB)	1 µl
Acc65I (NEB)	1 µl
Buffer R3.1 (NEB)	3 µl
Distilled water	To 50 µl

Put the mix at 37 °C for 16 hours and isolate the 6 kb band from gel.

STEP 2

Once the genomic insert has been selected, two oligos need to be ordered:
TOP: 5‘TCGACC’-[GENOMIC INSERT]-‘G’3
BOTTOM: 5‘GTACC’[GENOMIC INSERT REVERSE COMPLEMENT]-‘GG’3

Anneal the oligos according to the following protocol:

Component	Volume
TOP oligo (100 µM)	1 µl
BOTTOM oligo (100 µM)	1 µl
PNK (NEB)	1 µl
PNK buffer 10x (NEB)	1 µl
Distilled water	6 µl

**Total**	10 µl

Heat the mix to 95 °C for 3 minutes and ramp down to 20 °C at 5 °C / min.
Dilute the oligos 1:10 by adding 90 µl water to the mix.

STEP 3

Ligate the plasmid and annealed oligos in the following mix:

**Component**	**Volume**
Digested pmGFP-P2A-K0-P2A-RFP plasmid	30 ng
Annealed genomic insert	1 µl @ 1 µM
T4 DNA ligase (NEB)	1 µl
T4 DNA ligase buffer 10X (NEB)	1 µl
Distilled water	To 10 µl

Set at 21 °C for 60 minutes and transform.

Supplementary References:

1. Kim, H. K. et al. Predicting the efficiency of prime editing guide RNAs in human cells. *Nat. Biotech.* **39**,198–206 (2021).
2. Anzalone, A. V. *et al.* Search-and-replace genome editing without double-strand breaks or donor DNA. *Nature* **576**, 149–157 (2019).
3. Doench, J., Fusi, N., Sullender, M. *et al.* Optimized sgRNA design to maximize activity and minimize off-target effects of CRISPR-Cas9. *Nat Biotechnol* **34**, 184–191 (2016).
