# Supplementary material for: Mutation-specific reporter for optimization and enrichment of prime editing
Source: Nat Commun. 2022 Mar 1;13:1028. doi: 10.1038/s41467-022-28656-3 (PMC8888566; doi:10.1038/s41467-022-28656-3)
Supplement: Supplementary file 2 — Reporting Summary [file 41467_2022_28656_MOESM2_ESM.pdf]

nature research

Corresponding author(s): Sabine A. Fuchs

Last updated by author(s): 2-2-2022

Reporting Summary

Nature Research wishes to improve the reproducibility of the work that we publish. This form provides structure for consistency and transparency in reporting. For further information on Nature Research policies, see our Editorial Policies and the Editorial Policy Checklist.

Statistics

For all statistical analyses, confirm that the following items are present in the figure legend, table legend, main text, or Methods section.

n/a	Confirmed	
☐	☒	The exact sample size (*n*) for each experimental group/condition, given as a discrete number and unit of measurement
☐	☒	A statement on whether measurements were taken from distinct samples or whether the same sample was measured repeatedly
☐	☒	The statistical test(s) used AND whether they are one- or two-sided<br>*Only common tests should be described solely by name; describe more complex techniques in the Methods section.*
☐	☒	A description of all covariates tested
☐	☒	A description of any assumptions or corrections, such as tests of normality and adjustment for multiple comparisons
☐	☒	A full description of the statistical parameters including central tendency (e.g. means) or other basic estimates (e.g. regression coefficient) AND variation (e.g. standard deviation) or associated estimates of uncertainty (e.g. confidence intervals)
☐	☒	For null hypothesis testing, the test statistic (e.g. *F*, *t*, *r*) with confidence intervals, effect sizes, degrees of freedom and *P* value noted<br>*Give P values as exact values whenever suitable.*
☒	☐	For Bayesian analysis, information on the choice of priors and Markov chain Monte Carlo settings
☒	☐	For hierarchical and complex designs, identification of the appropriate level for tests and full reporting of outcomes
☒	☐	Estimates of effect sizes (e.g. Cohen's *d*, Pearson's *r*), indicating how they were calculated

Our web collection on statistics for biologists contains articles on many of the points above.

Software and code

Policy information about availability of computer code

Data collection	No software was used for data collection.
Data analysis	For comparisons of multiple groups, an ordinary one-way ANOVA with Holm–Sidak correction for multiple comparisons was used in Prism version 8.4.1 (GraphPad Software, LLC).<br>Flow cytometry data was analyzed using FlowJo. RNA sequencing was analyzed using DESeq2 in RStudio (Love, 2014), gene set enrichment analysis (Subramanian, 2005), and enrichR (Chen, 2013). All figures were made in Prism (GraphPad Software) or GGPlot2 (Wickham, 2012) in RStudio. Sanger sequencing was quantified using EditR (Kleusner, 2018) or TideR (Brinkman, 2018).

For manuscripts utilizing custom algorithms or software that are central to the research but not yet described in published literature, software must be made available to editors and reviewers. We strongly encourage code deposition in a community repository (e.g. GitHub). See the Nature Research guidelines for submitting code & software for further information.

Data

Policy information about availability of data

All manuscripts must include a data availability statement. This statement should provide the following information, where applicable:

- Accession codes, unique identifiers, or web links for publicly available datasets
- A list of figures that have associated raw data
- A description of any restrictions on data availability

High-throughput sequencing data have been deposited at the National Center of Biotechnology Information’s Sequence Read Archive database at PRJNA802707. The HTS data is included in Figures 1e, 3a, 3b, and Supplementary Figure 8c. The RNA sequencing data generated in this study have been deposited in the National

Center of Biotechnology Information (NCBI) database under accession code GSE195977. The RNA-sequencing data is presented in Figure 4 a-d and Supplementary Figure 10 b-d.

Field-specific reporting

Please select the one below that is the best fit for your research. If you are not sure, read the appropriate sections before making your selection.

☒ Life sciences ☐ Behavioural & social sciences ☐ Ecological, evolutionary & environmental sciences

For a reference copy of the document with all sections, see nature.com/documents/nr-reporting-summary-flat.pdf

Life sciences study design

All studies must disclose on these points even when the disclosure is negative.

Sample size	No sample-size calculation was performed. In experiments designed to compare the efficiency of specific prime editing designs or to compare the efficiency of prime editing across different conditions, sample sizes were chosen to reflect statistical differences in efficiency across 3 repeated experiments or 3 biologically indepedent replicates. Three replicates are sufficient to calculate statistical differences in this setting (also see Anzalone et al. Nature, 2019, Nelson et al. Nature Biotechnology, 2021).<br>For genomic and functional repair of biallelic CFTR-508del mutations, two intestinal organoid lines raised from rectal biopsies from two different donors were used. For genomic repair of biallelic ABCB4-E1012X mutations, one liver-derived organoid line from one patient was used. For genomic repair of biallelic ATP8B1-R600Q, one intestinal organoid line raised from rectal biopsies from one patient was used.
Data exclusions	No data was excluded from analysis.
Replication	No attempts at replication of experiments in the paper failed. Data in this paper was reproducible; numbers of experiments have been provided.
Randomization	In vitro cultures were paired and distributed into control and experimental prime editing design groups for comparison. Specifically, cell lines or organoid cells from each donor were harvested and pooled into one large pool before each transfection experiment. The different transfection conditions were applied to sub-pools of equal size, taken from this larger pool. Therefore, no bias existed in the allocation of experimental conditions.
Blinding	The analysis of FACS data was automated in FlowJo. The analysis of Sanger sequences was automated in Tide (Brinkman et al. 2014) or EditR (Kluesner et al. 2018). The analysis of NGS data was automated in RGEN PE-analyzer (Hwang et al. 2021). Therefore, blinding was not relevant for these analyses.

Reporting for specific materials, systems and methods

We require information from authors about some types of materials, experimental systems and methods used in many studies. Here, indicate whether each material, system or method listed is relevant to your study. If you are not sure if a list item applies to your research, read the appropriate section before selecting a response.

Materials & experimental systems

n/a	Involved in the study
☒	☐ Antibodies
☐	☒ Eukaryotic cell lines
☒	☐ Palaeontology and archaeology
☒	☐ Animals and other organisms
☐	☒ Human research participants
☒	☐ Clinical data
☒	☐ Dual use research of concern

Methods

n/a	Involved in the study
☒	☐ ChIP-seq
☐	☒ Flow cytometry
☒	☐ MRI-based neuroimaging

Eukaryotic cell lines

Policy information about cell lines

Cell line source(s)	HEK293T (ATCC), Caco-2 (ATCC), HeLa (ATCC)
Authentication	Cells were authenticated by the supplier using STR analysis
Mycoplasma contamination	All cell lines tested negative in repeated (3-monthly) mycoplasma contamination tests.
Commonly misidentified lines<br>(See ICLAC register)	None used.

Human research participants

Policy information about studies involving human research participants

Population characteristics	Healthy control organoid lines derived from human liver biopsies were obtained from anonymous donors (age and gender not disclosed).
Recruitment	Tissue biopsies from the liver of a patient with ABCB4 deficiency (PFIC3) was obtained during a liver transplant procedure in the UMCG, Groningen. Rectal biopsies used for intestinal organoid culture from a patient with ATP8B1 deficiency (PFIC1) were obtained at the outpatient clinic in the UMCU, Utrecht. Biobanked intestinal organoids are stored and cataloged (https://huborganoids.nl/) at the foundation Hubrecht Organoid Technology (http://hub4organoids.eu). All biopsies were used after written informed consent. All patient mutations were genetically verified. No self-selection or other biases are applicable given the genetic nature of diseases.
Ethics oversight	The study was approved by the responsible local ethics committees (Institutional Review Board of the University Medical Center Utrecht and University Medical Center Groningen (STEM: 10-402/K; TcBio 14-008; Metabolic Biobank: 19-489)). For cystic fibrosis organoids, collection of patient tissue and data was performed following the guidelines of the European Network of Research Ethics Committees (EUREC).

Note that full information on the approval of the study protocol must also be provided in the manuscript.

Flow Cytometry

Plots

Confirm that:

- [x] The axis labels state the marker and fluorochrome used (e.g. CD4-FITC).
- [x] The axis scales are clearly visible. Include numbers along axes only for bottom left plot of group (a 'group' is an analysis of identical markers).
- [x] All plots are contour plots with outliers or pseudocolor plots.
- [x] A numerical value for number of cells or percentage (with statistics) is provided.

Methodology

Sample preparation	1-6 days after transfection using the fluoPEER plasmid, HEK293T cells, Caco-2 cells, HeLa cells, and human intestinal- and liver derived organoid cells were dissociated with Trypsin or TrypLE for 2-3 minutes at 37°C. The cells were washed once using Advanced DMEM/F12 and resuspended in 400 µl FACS buffer (PBS with 2 mM EDTA and 0.5% BSA).
Instrument	Flow cytometry was performed on the FACS Fortessa (BD Biosciences) and sorting was performed on the FACS FUSION (BD Biosciences) using FACS Diva software (BD Biosciences).
Software	FlowJo™ Software was used for data analyses.
Cell population abundance	For HEK293T cells, typically 30-60% were living, GFP+ (transfected) cells. For organoid cells, typically 5-10% were living, GFP+ (transfected) cells. For Caco-2 and Hela cells, typically 15-25% were living, GFP+ (transfected) cells. The percentage of Cherry + cells (fluoPeer-edited) among GFP+ varried between conditions (0-80%), depending on the relative activity of prime editing.
Gating strategy	SSC/FSC and DAPI were used to identify live cells; GFP was used to identify cells transfected with the fluoPEER plasmid and cotransfected with prime editing machinery (PE2 + pegRNA plasmids and nicking sgRNA plasmid for PE3/PE3b conditions). Finally, the transfected cell population was selected based on a negative control sample without a fluorescent plasmid. The reporter-edited population was selected (GFP+Cherry+) based on 'fluoPEER plasmid only' conditions , and sorted for analysis of genomic editing. For the RNA-sequencing experiment, reporter-edited population was selected (GFP+Cherry+) and reporter-unedited (GFP+Cherry-) populations were selected based on 'fluoPEER plasmid only' conditions. For cell cycle analysis, 60 minutes before harvesting for FACS analysis, 10 µg/ml Hoechst 33342 (ThermoFisher) was added to the culture medium and gates for G1, S, and G2 phase were determined based on a a G2-phase control, which had been incubated with 200 ng/ml nocodazole for 20 hours.

- [x] Tick this box to confirm that a figure exemplifying the gating strategy is provided in the Supplementary Information.
