# Supplementary material for: Mutation-specific reporter for optimization and enrichment of prime editing
Source: Nat Commun. 2022 Mar 1;13:1028. doi: 10.1038/s41467-022-28656-3 (PMC8888566; doi:10.1038/s41467-022-28656-3)
Supplement: Supplementary file 3 — Description of Additional Supplementary Files [file 41467_2022_28656_MOESM3_ESM.pdf]

Title: Supplementary Data file 1:
Description: Differentially expressed genes in fluoPEER-edited vs. -unedited cells (included as a separate excel file)

Title: Supplementary Data file 2:
Description: Oligos used in this study (included as a separate excel file)
